# Cyanotoxins and the Nervous System

**DOI:** 10.3390/toxins13090660

**Published:** 2021-09-16

**Authors:** James S. Metcalf, Maeve Tischbein, Paul Alan Cox, Elijah W. Stommel

**Affiliations:** 1 Brain Chemistry Labs, Box 3464, Jackson, WY 83001, USA; paul@ethnomedicine.org; 2 Dartmouth-Hitchcock Medical Center, Department of Neurology, Geisel School of Medicine at Dartmouth, Lebanon, NH 03756, USA; maeve.tischbein@hitchcock.org (M.T.); Elijah.W.Stommel@hitchcock.org (E.W.S.)

**Keywords:** cyanobacteria, toxin, CNS, chronic, acute, neurodegeneration

## Abstract

Cyanobacteria are capable of producing a wide range of bioactive compounds with many considered to be toxins. Although there are a number of toxicological outcomes with respect to cyanobacterial exposure, this review aims to examine those which affect the central nervous system (CNS) or have neurotoxicological properties. Such exposures can be acute or chronic, and we detail issues concerning CNS entry, detection and remediation. Exposure can occur through a variety of media but, increasingly, exposure through air via inhalation may have greater significance and requires further investigation. Even though cyanobacterial toxins have traditionally been classified based on their primary mode of toxicity, increasing evidence suggests that some also possess neurotoxic properties and include known cyanotoxins and unknown compounds. Furthermore, chronic long-term exposure to these compounds is increasingly being identified as adversely affecting human health.

## 1. Introduction

Cyanobacteria are evolutionarily ancient photosynthetic organisms with fossils dating back 3.6 billion years [1]. Although previously referred to as blue-green algae, cyanobacteria are Gram-negative bacteria and have characteristics of such bacteria including the presence of lipopolysaccharide (LPS) in their cell wall [2]. They are common components of aquatic and terrestrial environments, and their growth is influenced by physical and chemical factors. In aquatic environments, conditions such as calm, sunny weather along with the provision of nutrients including nitrogen and phosphorous can result in mass accumulations of cyanobacteria, manifested as blooms and scums [3]. Often unsightly, and potentially containing compounds such as geosmin and methylisoborneol which give the water an unpleasant taste and odor, cyanobacteria can negatively affect the aesthetics of water [4]. As some cyanobacterial cells are capable of producing gas vesicles to provide buoyancy, this allows them to float to the surface resulting in the potential for exponential concentration factor increases in cell number. Consequently, under these conditions of positive buoyancy and the action of wind, scums resembling thick green paint can form on shorelines and in embayments [3]. Given that cyanobacteria are capable of producing a wide range of bioactive compounds, with many considered to be toxins (cyanotoxins), any toxins naturally present within these cyanobacterial cells are able to reach a concentration that may pose a risk to people and animals.

Although there is evidence that cyanobacteria have had the capacity to produce toxins for millions of years [5,6], the toxic effects of exposure to cyanobacteria have been known since at least the late 19th century, including reports and investigations into the toxicity of *Nodularia* scum in Australia [7]. Periodic mass mortalities of animals including dogs, birds and cows have occurred with exposure to cyanobacteria which are often considered to be the proximal cause, given the detectable presence of cyanotoxins in clinical materials (e.g., [8,9,10,11,12,13,14,15,16]). From such observations, detailed investigations into toxic compounds produced by cyanobacteria have resulted in the elucidation of a number of compounds of human and animal health concern. Through rigorous assessments of axenic and/or monocyanobacterial laboratory cultures, environmental collections and clinical materials from animal and human intoxications, new and emerging toxic compounds produced by cyanobacteria continue to be characterized.

Although animals account for the majority of reported cyanobacterial intoxications, human populations exposed to cyanobacterial toxins have also resulted in reported illness and death. Examples include: outbreaks of gastroenteritis from exposure to LPS [17,18], hepatomegaly from (presumed) exposure to cylindrospermopsin [19] and pneumonia-like symptoms in army cadets after oral exposure to *Microcystis* scum during aquatic drill exercises [20]. These and other cases highlight the potential risk of acute exposure to cyanobacterial toxins. The most high-profile case of human exposure to cyanobacterial toxins occurred in Caruaru, Brazil in 1996. At a haemodialysis clinic, 100 people died and 52 people had confirmed exposure to the hepatotoxic microcystins through intravenous administration of water ineffectively treated at the clinic and obtained from a lake known to harbor cyanobacterial blooms [21,22]. Later assessment and analysis of materials related to these intoxications determined that the cytotoxic cylindrospermopsins were also present [23]. During this poisoning event, many of the patients exposed to this ineffectively treated water complained of neurological symptoms such as tinnitus, dizziness, vertigo and vision issues [21,22]. This led to the idea that cyanotoxins not traditionally classified as being neurotoxic may demonstrate neurological effects and may negatively interact with the mammalian central nervous system (CNS). Therefore, it may be necessary to expand the known modes of action of cyanobacterial toxins to take into account other less-reported toxic effects. Consequently, this review aims to examine what is currently known about cyanotoxins that can affect the nervous system, particularly the CNS. 

## 2. Toxins That Affect the Brain and Nerves

**Figure 1:**
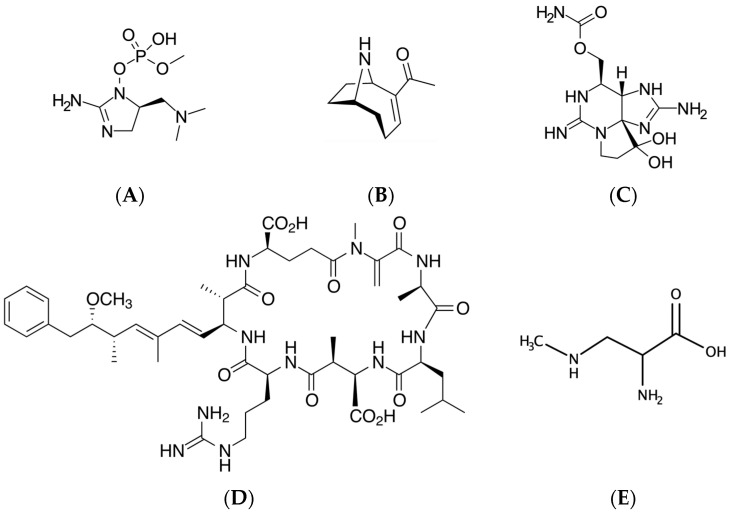
Structures of cyanotoxins with potential neurotoxicological implications. (**A**), guanitoxin; (**B**), anatoxin-a; (**C**), saxitoxin; (**D**), microcystin-LR; (**E**), β-N-methylamino-L-alanine.

Traditionally, cyanotoxins have been categorized with respect to what major aspects of mammalian physiology are adversely affected (e.g., cytotoxins, hepatotoxins and gastrointestinal toxins). Cyanobacterial neurotoxins (Figure 1) are an additional class of compounds with demonstrated neurological effects as the principal known mode of action [4]. However, other toxic cyanobacterial compounds not traditionally considered neurotoxins have been increasingly found to have neurological effects or are able to enter the CNS (e.g., [24]). Therefore, for the purposes of this review, cyanotoxins with neurotoxic effects have been divided into the following categories:

### 2.1. Traditional Acute Neurotoxins 

These are largely alkaloid or organosphosphorous compounds that are extremely potent in mammalian systems. Their effects are largely observed within minutes to hours. Although chronic, long-term damage cannot be excluded, if the victim survives initial intoxication (either naturally or through medical intervention), no long-lasting adverse health effects are currently known to occur. 

#### 2.1.1. Anatoxin-a and Homologues

This class of low molecular weight alkaloid toxins encompasses about 7 cyanobacterially-derived compounds and degradation products, which can be expanded through known synthetic analogs [25,26]. The two anatoxin-a variants produced by cyanobacteria are anatoxin-a and homoanatoxin-a [26]. They are fast acting, function as acetylcholine mimics that bind to nicotinic acetylcholine receptors and result in the inability of acetylcholine esterase to remove these toxins from this essential CNS receptor [27]. Subsequently, this results in continued excitation and depolarization of neurons and can cause paralysis, asphyxiation and death when exposed to a sufficiently high concentration. In the CNS anatoxin-a has been shown to affect dopamine [28], blood pressure and heart rate [29]. Furthermore, anatoxin-a has been attributed to an outbreak of acute human poisoning resembling a “cerebellar syndrome” (including ataxia, dizziness and visual disturbances) following consumption of tunicates in southern France [30,31]. To a lesser extent, anatoxin-a can affect muscarinic acetylcholine receptors [27]. Often produced by filamentous cyanobacteria such as *Phormidium* in benthic mats, when associated with the co-production of taste and odour compounds, anatoxin-a has resulted in the deaths of dogs who consume these mats, either from waterbodies or from filaments that adhere to the fur of the animals [14]. When birds are affected by anatoxin-a, one observed physiological response is opisthotonous, whereby the muscle at the back of the neck is affected and contracts, resulting in the head lying along the back of the bird [26,32].

#### 2.1.2. Saxitoxins

Traditionally considered a product of marine dinoflagellates, saxitoxins are a group of 57 guanidinium alkaloids that are responsible for paralytic shellfish toxin poisoning in marine environments, largely through the consumption of contaminated shellfish [33,34]. The saxitoxins themselves are further divided, dependent on their chemical structures and substitutions, into classes such as C toxins, G toxins and LW toxins [33,35]. Of the acute cyanobacterial neurotoxins, saxitoxins have been known to cause human deaths as a result of their production by marine dinoflagellates which then contaminate shellfish with this highly potent neurotoxin [36]. Saxitoxins can be produced by cyanobacterial genera including *Aphanizomenon*, *Dolichospermum* and *Cylindrospermopsis* and blooms of these organisms have the potential to be highly toxic to animals and humans [4]. The majority of saxitoxin variants produced by cyanobacteria include saxitoxin and neosaxitoxin, in addition to LW (*Lyngbya wollei*) toxins [35]. Saxitoxins act by blocking voltage-gated sodium channels [37] and if present in sufficiently high concentration can result in paralysis and death. Other neuronal channels such as potassium and calcium channels may also be affected by saxitoxins [38].

#### 2.1.3. Anatoxin-a(S) (Guanitoxin)

A naturally occurring organophosphate, anatoxin-a(*S*) has been associated with *Anabaena* (*Dolichospermum*) blooms, particularly in Danish lakes where it has resulted in the deaths of waterfowl [12]. Now renamed guanitoxin [39] and similar in structure to organophosphate pesticides and insecticides, this toxin is able to inhibit acetylcholine esterase [39,40,41,42], an essential enzyme that removes acetylcholine from the synapse in mammalian neurons. Consequently, inactivation of this enzyme by guanitoxin can result in paralysis and asphyxiation and is also used in vitro as a diagnostic test for its presence [40]. Using such an assay, its presence has been inferred in desert crust assemblages of cyanobacteria largely comprised of the genus *Microcoleus* [43]. Although not common in terrestrial environments, intoxications of dogs that drink water overlying these desert crusts have been observed [16]. Although originally similar in name to anatoxin-a, guanitoxin is dramatically different to anatoxin-a and, as with other organophosphates, poisoning results in hypersalivation, as denoted by *S* in the original name for guanitoxin [39,42].

### 2.2. Neurotoxins Associated with Neurodegeneration

As described above, acute exposure to certain cyanobacteria and their toxins is relatively well known. However, there is also on-going research investigating the long-term effects and/or chronic exposure to cyanobacterial toxins. For example, there is increasing evidence that microcystins, nodularin and cylindrospermopsin all have potential deleterious effects with respect to tumor promotion and cancer after exposure to concentrations that in the short term are unlikely to result in illness (e.g., [44,45,46,47]). Many parts of the human body are able to deal with toxicants through dilution (cell division), detoxication and metabolism. However, the CNS contains neurons, which are large, non-dividing cells with substantial metabolic demands and may accumulate damage with age [48] and encountered insults. Consequently, this population of highly specialized cells may be particularly susceptible to the long-term actions of low concentrations of toxic compounds. To date, there are several cyanobacterial toxins that have been associated with neurodegeneration. 

#### 2.2.1. BMAA and Isomers

Increasingly, neurodegenerative diseases such as amyotrophic lateral sclerosis (ALS; also known as motor neurone disease) are thought to have an environmental component and do not arise as a consequence of genetics alone [49]. Thus, studying compounds associated with and identified as risk factors for these diseases may lead to clues concerning disease etiology and treatments. One particular disease and case study is ALS/Parkinsonism dementia complex (ALS/PDC), which was at one time known to be prevalent on the island of Guam [50]. Analysis of various factors associated with ALS/PDC identified diet as an important correlate in terms of developing this disease [51]. Further analyses of indigenous diets found that certain popular foodstuffs were high in a novel amino acid called β-*N*-methylamino-L-alanine (BMAA) that, when fed to chicks, resulted in neurotoxicity [52,53]. Current research suggests that BMAA can be produced by cyanobacteria [54,55], marine [56] and freshwater [57] diatoms and some chemoheterotrophic bacteria, such as members of the genus *Paenibacillus* spp. [58]. Increasingly the link between BMAA detection and the occurrence of dinoflagellates in marine waters suggests that this group of potentially harmful photosynthetic algae may also be capable of producing BMAA [59,60].

Although Guam was a focus for BMAA and ALS/PDC, the examination of Guamanian and non-Guamanian human brains showed that BMAA was present in other geographic locations [61,62]. Moreover, the work of Pablo and colleagues also showed that this amino acid is associated with additional neurodegenerative diseases such as ALS and Alzheimer’s disease (AD) [62]. Intriguingly, experiments feeding non-human primates with BMAA showed the development of hallmarks of human neurodegenerative diseases such as β-amyloid plaques and neurofibrillary tangles (hallmarks of AD as well as Guamanian ALS/PDC) as well as other neuropathological features (e.g., microglial activation) that are consistent with diseases such as ALS and AD [63,64]. While it is currently difficult to definitively determine how significant BMAA exposure is as a causal factor in ALS or AD, BMAA has been increasingly implicated as a risk factor for human neurodegenerative disease and requires greater attention [65,66].

Including BMAA, four naturally occurring isomers are currently considered to exist. Studies using the isomers *N*-(2-aminoethyl)glycine (AEG) and 2,4-diaminobutyric acid (DAB) have shown these compounds to possess neurotoxicity. Within the CNS, AEG may potentially act upon different receptors than BMAA and, in some cases, be considered more toxic [67,68]. Although BMAA exposure may occur throughout the world, e.g., through the consumption of contaminated shellfish [69], further research is needed to understand the occurrence and toxicity of all these neurotoxic isomers as well as more fully determine the risk of exposure and their adverse effects on neurological health. Moreover, as these neurotoxic amino acids can co-occur in harmful algal and cyanobacterial blooms, such exposure scenarios raise additional concerns regarding the synergistic effects of cyanotoxins [70].

#### 2.2.2. Aetokthonotoxin

The interaction of humans and animals with the environment has the potential for new and emerging diseases and toxicoses to be observed and reported. One example is the identification of avian vacuolar myelinopathy (AVM) in the brains of birds, particularly eagles that feed near lakes [71,72]. Certain lakes are known to be sites where outbreaks of this disease occur and, until recently, no known toxicants have been definitively identified. The disease was associated with exposure to *Hydrilla*, and epiphytic cyanobacteria present on the surface of this plant were considered as potential causative agents [73]. Feeding test animals extracts of certain collections of *Hydrilla* resulted in gross pathology consistent with AVM, and epiphytic cyanobacteria were also shown to be toxic to bird species such as coots that were fed these extracts [74]. Extensive research has now identified a brominated cyanobacterial toxin, aetokthonotoxin, which results in the production of vacuoles and lesions in the brains of birds fed this toxin, consistent with it being a cause of AVM [74].

### 2.3. Cyanotoxins with Potential Neurological Effects

#### 2.3.1. Microcystins

Microcystins are common cyclic peptides that are known to be produced by cyanobacterial genera such as *Microcystis*, *Anabaena* and *Planktothrix* [4]. They are comprised of 7 amino acids and due to substitutions and modifications of the structure, over 240 variants are currently known, each with differing toxicities [75,76]. Exposure to microcystins has resulted in the deaths of animals such as cows and dogs and are one of only a few cyanotoxins that have regulations for permissible concentrations in drinking and recreational waters [8]. Although traditionally classified as a hepatotoxins, people who were exposed to microcystins complained of neurological symptoms such as tinnitus and vision problems, leading to the idea that microcystins may possess adverse neurological activities [21,22,23,24]. 

Through investigations examining the hepatotoxic effects of microcystins, one molecular mode of action is the inhibition of protein phosphatases and phosphoprotein phosphatases [77,78]. Protein phosphatases are key cellular enzymes that are also found within the CNS and their inhibition has been implicated in the development of neurodegenerative diseases [79]. Although the permissible concentrations of microcystins in drinking and recreational waters include the potential for tumor promotion, current and future determinations of permissible microcystin concentrations are considering their potential for neurotoxicity [80].

#### 2.3.2. Lipopeptides

Assessment of *Lyngbya majuscula* (*Moorea producens*) has shown that this marine cyanobacterium is capable of producing a wide range of bioactive compounds with unusual and unique toxicological actions [81]. A number of lipopeptides that contain an aliphatic group can be produced by cyanobacteria and are capable of affecting voltage gated sodium channels on neurons. Using N-methyl-D-aspartate (NMDA) agonists, the action of antillatoxins and kalkitoxin on cerebellar granule cells could be prevented [82]. Further differences have been observed between the lipopetides with antillatoxins having the potential to activate voltage gated sodium channels, whereas jamaicamides and kalkitoxin inhibit these channels, in a manner similar to saxitoxin [25,83,84]. The assessment of *L. majuscula* shows the importance of assessing cyanobacteria from a wide range of environments for compounds with toxic and therapeutic potential as such voltage-gated sodium channel inhibitors may be applicable as analgesics [85,86]. 

#### 2.3.3. Cylindrospermopsin

Although traditionally considered a cytotoxin, cylindrospermopsin was identified following an assessment of cyanobacterial bloom material from a human poisoning incident on Palm Island, Australia [87,88]. Here, an outbreak of hepatic gastroenteritis resulted in the hospitalization of 140 people, with some showing an enlarged liver (hepatomegaly) [87]. Several studies have assessed the neurotoxic potential of this cyanotoxin and some of the responses seen could be a consequence of the general cytotoxic action of cylindrospermopsin, e.g. affecting reactive oxygen species and DNA damage (reviewed by [89]). Using extracts and purified fractions from *Cylindrospermopsis raciborskii*, Kiss et al. [90] showed a neuroactive effect of these preparations on snail neurons, although Vehovsvky et al. [91] considered that this effect was due to an anatoxin-a-like compound. Using cell lines, Tasker et al. [92] showed cylindrospermopsin-induced apoptosis and inflammation in murine neuroblastoma and glial cells.

### 2.4. Other Cyanobacterial Compounds of Possible Neurotoxicological Interest

In addition to the known cyanobacterial toxins, these photosynthetic Gram-negative organisms are capable of producing a plethora of bioactive substances [81]. Many of them are capable of inhibiting a variety of eukaryotic enzymes such as elastase, trypsin and chymotrypsin (reviewed by [81]). Of potential interest to neurotoxicity are compounds such as Anabaenopeptin F, Oscillamides B and C and cyanostatins A and B, which have the potential to inhibit protein phosphatases [93,94], enzymes implicated in the development of neurodegenerative disease [79] and one of the proposed mechanisms of microcystin neurotoxicity [24]. Other aspects of neurology that may be influenced by cyanobacterial compounds include Anabaenopeptin F and biogenic amines that may influence or mimic the effect of norepinephrine [81]. Nodularin is potentially a compound of interest and has been associated with both human and animal poisonings. Although there is limited neurotoxic evidence, nodularin is structurally and toxicologically similar to microcystin, a cyanotoxin with likely neurotoxic properties [24]. A component of the outer membrane of Gram-negative bacteria, LPS is a known gastro-intestinal irritant which has been implicated as the causative agent of a number of human poisoning incidents [2]. Furthermore, as it is a component of the outer membrane of Gram-negative bacteria then chemoheterotrophic bacteria are also capable of producing this complex. Although compositions of LPS can differ between chemoheterotrophic bacteria and cyanobacteria [2,95] and it is not a known neurotoxin, some reports have shown that *E. coli* LPS can induce the formation of lipocalin-2, affecting the expression of chemokines in the CNS which may then affect cell migration and inflammation [96].

## 3. Transportation into the CNS

When the blood–brain barrier (BBB) is intact, then transporters may be required to allow compounds, such as cyanotoxins, to enter the CNS. However, if the BBB is damaged, then this would likely increase the range of compounds that could enter the CNS and cause adverse effects [97]. Preventing cyanotoxins from entering the CNS may serve as a therapeutic target during intoxications. Although this strategy may have little effect on acute intoxications, where high toxin concentrations rapidly affect the CNS, such research may benefit those who are chronically exposed to cyanotoxins as a means to prevent adverse, long-term health effects. 

In addition to identifying transporters for individual cyanotoxins, likely CNS transporters might be inferred from the examination of structurally and toxicologically related compounds. Anatoxin-a has been shown to be synthesized from cocaine [27], an alkaloid that displays neurological effects and, with long-term use, is associated with cognitive decline [98]. Although anatoxin-a has been shown to cause a release of dopamine, rather than affecting dopamine and serotonin receptors such as with cocaine, anatoxin-a is an acetylcholine mimic [99,100]. The most common acetylcholine mimic people are exposed to is nicotine and anatoxin-a affects nicotinic and to some extent muscarinic acetylcholine receptors [101]. Nicotine is able to enter the CNS extremely quickly, as evidenced by inhalation of tobacco combustion products. It is most likely transported by cation/organic molecule antiporter systems, which can be prevented by drugs such as verapamil and clonidine [102]. Although anatoxin-a can affect muscarinic acetylcholine receptors, muscarine is unable to cross the BBB and these receptors are found in many mammalian organs [103]. 

Guanitoxin is the only known naturally-occurring organophosphate molecule and is occasionally found in cyanobacterial blooms and potentially terrestrial assemblages of cyanobacteria [12,43]. Synthetic organophosphates have been widely used as pesticides and insecticides for many years and have been linked to neurodegenerative diseases such as Parkinson’s disease (PD) and ALS. A wide range of such compounds are known and used including malathion, parathion and chlorpyrifos. They are effective as they inhibit acetylcholine esterase, an essential enzyme that removes acetylcholine from synapses [104]. Although unknown for exposure to guanitoxin, long-term exposure to some organophosphates has been linked to the development of cancer in farmers including bladder, prostate, kidney and lung cancer [105]. Given that organophosphates have been linked to both PD [106,107,108] and ALS [109,110], it is plausible that guanitoxin may exhibit chronic effects at relatively low concentrations. Moreover, certain organophosphates can induce a form of neurotoxicity, known as organophosphate-induced delayed polyneuropathy (OPIDP) [111]. There have been several occurrences of human poisonings resulting in OPIDP, mostly involving tri-*ortho*-cresyl phosphate, which functions as a weak AChE inhibitor [111]. Whether guanitoxin could lead to OPIDP is not known, but the mechanistic parallel and potential for human harm warrants investigation. Certain organophosphates such as soman, sarin, chlopyrifos and malathion are known to break down the BBB [112,113,114], thereby allowing entry of these and/or other compounds into the CNS where they are then capable of disrupting neuronal processes. 

Saxitoxins are extremely potent inhibitors of voltage-gated sodium channels [33]. They are capable of crossing the BBB [115,116] and increase serotonin concentrations in rats at high doses [117]. However, the concentration to which an organism is exposed may determine whether there is sufficient saxitoxin biologically available to cross the BBB, as a result of its rapid binding to voltage-gated sodium channels throughout the body. Tetrodotoxin, another potent toxin that acts on voltage-gated sodium channels may provide insight as to the pharmacological action of saxitoxins, although studies so far indicate that tetrodotoxin is unable to cross the BBB [118]. Research suggests that long-term exposure to saxitoxins may cause adverse health effects on antioxidant systems and DNA, as demonstrated in fish and mammalian models [34].

Although microcystins are primarily considered to be hepatotoxins and tumor promoters, their potential neurotoxicity is receiving increasing attention. In terms of CNS transport, multi-organ organic anion transport system (OATP) proteins have been implicated as allowing compounds to cross the BBB and, of this large family of transport proteins, human OATP1A2 may transport microcystins [119,120,121]. Although further research is required to understand whether this happens in the human brain, Furstein et al. [121] showed that differences in transport can be observed, where microcystin-LF may be more efficiently transported than the more hydrophobic, microcystin-LR. This observation is in keeping with the potential differences in toxicity between microcystin variants and their hydrophobicities [122].

Cylindrospermopsins are another class of cyanobacterial toxins that may also have neurological implications [89], with inflammatory effects observed in microglial and neuroblast cell lines [92] and neurotoxicity observed in tilapia [123]. Although there are no known transporters for cylindrospermopsins across the BBB or evidence of passive diffusion [124], enzyme-linked immunosorbent assay (ELISA) analysis of fish brains has shown the presence of this neurotoxin. However, as false positives can be observed with commercial cylindrospermopsin ELISAs [125], further research on the transportation and presence of this cyanotoxin in the brain are required.

The neurotoxic amino acid, BMAA, is known to accumulate in the brain after exposure [126] and has been found in human and dolphin brains [62,127]. Experiments concerning potential transporters identified the large neutral amino acid transporter [128], as this amino acid is uncharged at physiological pH [129]. Furthermore, this transporter is considered to be specific for the L-enantiomer of BMAA. Although D-BMAA has been found in the mammalian brain, this compound is currently considered to be an enzymatic product formed within the CNS [67]. Given that essential amino acids require transportation across the BBB and that there are over 800 non-protein amino acids [130], is it conceivable that other non-incorporated, biologically active amino acids may also enter the CNS and have deleterious effects [131].

The identification of biologically active compounds in cyanobacteria continues to increase, through the identification of new classes of toxins and unique variants within a class. Included are many lipopeptides such as jamaicamides and antillatoxins that have neurological effects such as binding to sodium channels [84]. Although such channels are found throughout the peripheral nervous system (PNS) and CNS, further research may identify additional channels or transporters that allow them entry into the mammalian CNS.

## 4. Effects within the Peripheral and Central Nervous Systems

The compounds produced by cyanobacteria with neurotoxic potential are extremely diverse (Figure 1). However, such compounds generally affect essential systems within the cell. As previously mentioned, neurons represent a unique cell population in the CNS, and it is likely that certain neurons are particularly sensitive to cyanotoxin-induced damage and/or disruptions in cellular processes. Such convergent avenues for cyanotoxin-mediated cellular insults include:

### 4.1. Blocking of Essential Channels and Proteins

Compounds that block proteins and channels often result in rapid and severe effects in mammals. This mechanism is commonly observed for neurotoxins as well as frequently seen in acute intoxications. Cyanobacteria are capable of producing a range of protein blocking proteins such as estrogenic compounds and endocrine disruptors [81]. With respect to neurotoxicity, saxitoxins, antillatoxins and jamaicamides as well as non-cyanobacterial toxins such as tetrodotoxin, can block voltage-gated sodium channels resulting in a rapid inability to perform many neurological functions such as breathing [25,84,118,132]. Anatoxin-a binds to acetycholine receptors in competition with nicotine [133] and BMAA has the capacity to bind to NMDA and glutamate receptors [79,134,135]. 

### 4.2. Enzyme Inhibition

The screening of cyanobacteria has shown that small molecule eukaryotic enzyme inhibitors are common and such compounds include aeruginosins, anabaenopeptins, cyanopeptolins, microginins and microviridins (reviewed by [81]). A number of cyanobacterial toxins have been shown to be enzyme inhibitors, often of essential enzymes within mammalian cells. Microcystins (and nodularins) are able to inhibit protein phosphatases and phosphoprotein phosphatases [77,78], and this inhibition can be reversible or irreversible depending on the structure of the microcystin or nodularin variant [136]. Such phosphatases are key cell cycle enzymes and, as these enzymes are located within the CNS, any inhibition may lead to neurotoxic effects [24]. Guanitoxin is an inhibitor of acetylcholine esterase, an essential neurological enzyme. Such enzymes are found within the CNS and remove acetylcholine from synapses [40]. Similarly, with protein phosphatases, inhibition of acetylcholine esterase could result in neurotoxicity [42].

### 4.3. Protein Damage

For proper cell function, the correct sequence and folding of proteins is required. If this process is affected, then significant deleterious effects can ensue. As a naturally-occurring non-protein amino acid, BMAA can enter cells and replace the non-essential amino acid L-serine in proteins [137]. Consequently, such misfolded proteins (even at low rates of amino acid misincorporation) may then act incorrectly within the cell and give rise to the potential for long-term damage. This scenario is especially relevant to the CNS where the reduction in toxic burden through dilution and cell-division is difficult [138].

## 5. Natural Intoxication Events and Methods for Cyanotoxin Evaluation

In order to understand whether a cyanotoxin may have neurological and/or neurotoxicological effects, appropriate bioassays need to be performed. In the case of known neurotoxins (e.g., saxitoxin and anatoxin-a), established in vitro and in vivo tests exist [139]. These include binding to acetylcholine receptors for anatoxin-a [25], acetylcholine esterase inhibition assays for guanitoxin [40] and saxiphilin binding assays for saxitoxins [140]. As the structures of many of the acutely toxic cyanobacterial toxins are known, ELISAs are increasingly being developed to screen for their presence in environmental and clinical matrices [141]. However, when required and during intoxication events, confirmatory methods such as mass spectrometry should also be used. Although there are many mass spectrometry methods to evaluate known neurotoxins such as anatoxin-a and saxitoxin (e.g., [142]), only one method has been developed for guanitoxin using hydrophilic interaction liquid chromatography (HILIC)–mass spectrometry [143,144]. The further development of such methods is essential. For example, they allow for the detection of specific neurotoxins when investigating human and animal health incidents and can confirm the results of high-throughput screening methods.

Beyond the detection of cyanotoxins by physicochemical methods such as mass spectrometry (Table 1), additional assays may also be necessary to understand cyanotoxin exposure and toxicity, as well as to determine what components of the mammalian cell are affected (e.g., enzymes or proteins). For example, while hepatocytes can be used to understand the hepatotoxicity of microcystins, assessments using neurological cell lines have further revealed the inhibition of enzymes that could explain microcystin neurotoxicity [120,121]. Historically, the mouse bioassay has been useful for assessing alkaloid cyanotoxins (e.g., [145]), deriving guidance through LD_50_ values and, in the case of guanitoxin, for observing hypersalivation and lachrymation [25]. However, due to ethical considerations, mouse model alternatives should be sought where possible. Invertebrate bioassays represent one useful alternative as they are highly sensitive to cyanotoxins and, due to increased numbers of test organisms, may also allow for robust statistical assessments. Examples of non-mammalian bioassays include the evaluation of anatoxin-a and saxitoxin using invertebrates such as *Artemia salina* [146] or *Daphnia* spp. [147].

Natural poisoning events involving acutely neurotoxic compounds have been demonstrated multiple times (Table 1). The poisoning of domesticated animals such as dogs from anatoxin-a [14] or guanitoxin poisoning of birds that consume *Anabaena* bloom material [12], all demonstrate the importance of bioassays. Although cyanotoxins may be relatively easily identified from such events, understanding the long-term issues of exposure is more difficult. Therefore, the selection of an appropriate assay and/or system is an essential part of understanding the relationship between cyanotoxin exposure and potential disease occurrence. Furthermore, for understanding long-term exposures, extensive research and statistical analyses are often required. For example, the relationship between microcystin exposure and primary liver cancer was strengthened by analyzing microcystin concentrations in surface *versus* well water in China. Ueno et al. [46] showed that people who predominantly drank from well water were at lower risk of developing primary liver cancer than those who drank from surface waters potentially containing cyanobacteria. A similar relationship was observed in Eastern Europe with higher incidences of primary liver cancer in people who consumed water prepared from lakes with a history of known cyanobacterial blooms [148].

When used to complement epidemiological research, bioassays and model systems are fundamental to our understanding of natural intoxications or long-term exposures. The most recent example of long-term exposure to cyanobacterial toxins concerns BMAA, which was shown to be produced by freshwater and marine cyanobacteria and diatoms [54,56,57,59,170]. Through an assessment of locations with a high incidence of neurological disease, and combinations of specific neurological diseases such as ALS/PDC on the Island of Guam, BMAA and isomers have been identified as contaminating the traditional diet of Chamorro villagers [171]. Over 50 years of research on BMAA has shown that this amino acid is neurotoxic, present in the environment (including ancient pristine environments) and can be identified in brains of people who died of neurological illness [54,61,62,172,173]. Although these various lines of evidence are compelling in understanding the association of BMAA exposure and neurological disease, the biological assessment of BMAA toxicity has employed a wide variety of organisms including mice, rats, invertebrates and plants. Due to the wide range of toxic mechanisms ascribed to BMAA such as excitotoxicity, toxic metabolic products and receptor binding, many assays and systems have shown deleterious effects following exposure to this compound (reviewed by [174]). Of the mechanisms known, protein misincorporation is one possibility that could explain the development of neurological diseases such as ALS after BMAA exposure [175]. To this end, studies using non-human primates have shown that neuropathologies consistent with neurological diseases, including β-amyloid plaques, neurofibrillary tangles and microglial activation can be created after oral exposure [63,64]. 

Finally, another aspect of chronic cyanotoxin exposure is that often the effect of the purified toxin will be different or only partially account for the effect of the cyanobacterial extract containing the cyanotoxin of interest (e.g., [176]). Therefore, when assessing the potential for neurotoxicity some consideration of the chemical complexity of the extract may need to be considered.

## 6. Exposure Routes

Although the BBB is essential to protecting the CNS from pathogens and toxins [97], the actual route of cyanotoxin exposure may influence the toxicological outcome. Although much is known about oral exposure through drinking water and contaminated food such as shellfish and dietary supplements, other routes of exposure include medicinal water (e.g., haemodialysis) and recreational exposure through practices such as water sports, showering and bathing, the washing of utensils and other personal objects in contaminated water and skin irritation [4]. During episodes of acute exposure, human health can be protected through practices including remediation measures such as drinking water treatment and the provision of alternative water sources to affected consumers. However, concerning chronic exposure, the various media and the durations of exposure when defining safe cyanotoxin concentrations is much more difficult. Although this has been carried out regarding tumor promotion in the case of microcystin-LR in drinking water (e.g., [177]), potential neurotoxic effects of compounds not classically considered to be neurotoxins may also need to be taken into consideration. Subsequently, such findings may affect future drinking water and bathing Guideline Values.

Increasingly, inhalation is being considered as a major exposure route. Application of cyanotoxins as sprays into the nose of experimental animals has indicated that this may increase the toxicity of certain toxins such as microcystins and anatoxin-a [178]. Furthermore, toxin analyses have shown that microcystins, BMAA and anatoxin-a can be recovered from filter material used in environmental and personal air sampling devices [179,180,181,182], and that the presence of BMAA, microcystins and potentially guanitoxin in desert crust may become airborne during dust storms [43,183]. Such studies highlight the potential importance of this exposure route. Due to the proximity of the nasal cavity to the human blood stream and brain, if cyanotoxins are inhaled then this may result in a more rapid uptake of cyanotoxins into the CNS, as they can travel along the olfactory nerve and bypass the BBB [184].

## 7. Synergism and Co-Exposure—Could This Be Significant?

In vitro investigations into the toxicity of cyanobacterial extracts containing known toxins suggest that compounds or components of these extracts can interact. As such, questions concerning the synergism and antagonism of toxins in cyanobacterial extracts need to be considered (e.g., [185]). Although acute neurological intoxications are unlikely to be affected by minor toxic components that are present within extracts, long-term exposure to such compounds may have an altered outcome due to synergistic and antagonistic interactions. As cyanobacterial compounds are rarely found in isolation, especially in complex media such as water or air, interactions are likely to occur. In addition to the presence of different cyanobacterial toxins such as LPS, hepatotoxins, cytotoxins and neurotoxins, other toxicants such as microorganisms, viruses, metals, pesticides, persistent organic pollutants, nanoparticles and plastics, as examples, may significantly affect the toxicological outcome of the cyanobacterial bloom material [70]. Ultimately, complex analyses of air and water are required in order to determine what combinations of toxicants are present. Multi-factorial toxicity assessments (e.g., [186]), using a variety of cell types and organisms will then allow the determination of what interactions may take place, in addition to providing information concerning what the permissible concentrations of cyanotoxins may be under various circumstances.

## 8. Future Needs and Requirements

Increasingly, evidence suggests that cyanotoxins not traditionally considered neurotoxic as well as cyanobacterial extracts that do not contain known cyanotoxins can exhibit PNS and CNS toxicity. To fully understand their neurotoxicity, assessments of cyanobacterial extracts may need to include such neurotoxic outcomes when both devising bioassays and when selecting appropriate cell lines and organisms. Furthermore, when future guidelines are derived, such neurotoxic properties may need to be taken into consideration in order to protect human health.

Historically, poisoning events, both animal and human have driven research into understanding the toxicity of cyanobacteria. Increasingly, newly discovered compounds are found to have proven effects on neurological systems and the deleterious effects of other known compounds are being reviewed. Further analysis of human neurological disease cases occurring in proximity to harmful cyanobacterial and algal blooms may also be needed to fully consider their association. Ultimately, vigilance from medical practitioners and communication concerning the possible risks of cyanobacterial and algal blooms will help to protect human and animal health from the neurotoxicological effects of cyanobacteria.

## 9. Conclusions

Cyanobacteria are capable of producing a wide range of compounds with adverse effects in eukaryotic systems. Although toxins have traditionally been categorized based upon their primary mode of action, increasingly an ability of these compounds to have additional effects on neurological systems, of both the central and peripheral nervous systems is being recognized. Furthermore, the potential for cyanotoxins to have long-term chronic health effects is being acknowledged, in addition to the potential for synergism with anthropogenic and natural products, of cyanobacteria and contributed by other organisms in the environment. 

## Figures and Tables

**Table 1:** Aspects of known cyanotoxins with neurological implications.

Toxin	Mechanism of Action	Poisoning Examples	Detection Methods	References
Saxitoxins	Inhibition of voltage gated sodium channels	h, d	LC-FD, ELISA, LC-MS	[149,150,151,152,153,154]
Microcystins	Inhibition of protein phosphatases	h, c, f, b, d	ELISA, EIA, LC-PDA, LC-MS	[76,155,156,157,158,159,160]
Anatoxin-a	Acetylcholine mimic	d, b	ELISA, LC-PDA, LC-MS, EIA	[43,158,161,162,163,164,165]
Guanitoxin	Acetylcholine esterase inhibitor	b, d	EIA, LC-MS	[12,16,42,144]
BMAA	Protein misincorporation, inhibition of protein phosphatase	h, do	LC-FD, LC-MS	[61,62,79,137,166]
Cylindrospermopsins	Protein synthesis inhibitor	h, c	ELISA, EIA, LC-PDA, LC-MS	[10,23,87,125,167,168,169]

h, human; b, birds; d, dogs; c, cattle; f, fish; do, dolphins; ELISA, enzyme-linked immunosorbent assay, EIA, enzyme inhibition assay; LC-FD, liquid chromatography–fluorescence detection; LC-PDA, liquid chromatography–photodiode array detection; LC-MS, liquid chromatography–mass spectrometry.

## References

[1] Schopf J.W. (2010). The paleobiological record of photosynthesis. Photosynth. Res..

[2] Monteiro S., Santos R., Blaha L., Codd G.A., Meriluoto J., Spoof L., Codd G.A. (2017). Lipopolysaccharide endotoxins. Handbook of Cyanobacterial Monitoring and Cyanotoxin Analysis.

[3] Fogg G., Stewart W.D.P., Fay P., Walsby A.E. (1973). The Blue-Green Algae.

[4] Metcalf J.S., Codd G.A., Whitton B.A. (2012). Cyanotoxins. Ecology of Cyanobacteria II: Their Diversity in Space and Time.

[5] Rantala A., Fewer D.P., Hisbergues M., Rouhiainen L., Vaitomaa J., Börner T., Sivonen K. (2004). Phylogenetic evidence for the early evolution of microcystin synthesis. Proc. Natl. Acad. Sci. USA.

[6] Murray S.A., Mihali T.K., Neilan B.A. (2011). Extraordinary conservation, gene loss, and positive selection in the evolution of an ancient neurotoxin. Mol. Biol. Evol..

[7] Francis G. (1878). Poisonous Australian lake. Nature.

[8] Codd G.A., Lindsay J., Young F.M., Morrison L.F., Metcalf J.S., Huisman J., Matthijs H.C.P., Visser P.M. (2005). Harmful Cyanobacteria: From Mass Mortalities to Management Measures. Harmful Cyanobacteria.

[9] Mez K., Beattie K., Codd G., Hanselmann K., Hauser B., Naegeli H., Preisig H. (1997). Identification of a microcystin in benthic cyanobacteria linked to cattle deaths on alpine pastures in Switzerland. Eur. J. Phycol..

[10] Saker M.L., Thomas A.D., Norton J.H. (1999). Cattle mortality attributed to the toxic cyanobacterium *Cylindrospermopsis raciborskii* in an outback region of North Queensland. Environ. Toxicol..

[11] Mahmood N.A., Carmichael W.W., Pfahler D. (1988). Anticholinesterase poisonings in dogs from Cyanobacterial (Blue-Green Algae) Bloom dominated by *Anabaena flos-aquae*. Am. J. Vet. Res..

[12] Henriksen P., Carmichael W.W., An J.S., Moestrup O. (1997). Detection of an anatoxin-a(s)-like anticholinesterase in natural blooms and cultures of Cyanobacteria/blue–green algae from Danish lakes and in the stomach contents of poisoned birds. Toxicon.

[13] Pybus M.J., Hobron D.P., Onderka D.K. (1986). Mass Mortality of Bats Due to Probable Blue-green Algal Toxicity. J. Wildl. Dis..

[14] Codd G.A., Edwards C., Beattie K.A. (1992). Fatal attraction to cyanobacteria?. Nature.

[15] Wood S.A., Selwood A.I., Rueckert A., Holland P.T., Milne J.R., Smith K.F., Smits B., Watts L.F., Cary C.S. (2007). First report of homoanatoxin-a and associated dog neurotoxicosis in New Zealand. Toxicon.

[16] Chatziefthimiou A.D., Richer R., Rowles H., Powell J.T., Metcalf J.S. (2014). Cyanotoxins as a potential cause of dog poisonings in desert environments. Vet. Rec..

[17] Lippy E.C., Erb J. (1976). Gastrointestinal illness at Sewickley, PA. J. AWWA.

[18] Teixeira M.G., Costa M.C., de Carvalho V.L., Pereira M.S., Hage E. (1993). Gastroenteritis epidemic in the area of Itaparica Dam, Bahia, Brazil. Bull. Pan Am. Health Organ..

[19] Griffiths D.J., Saker M.L. (2003). The Palm Island mystery disease 20 years on: A review of research on the cyanotoxin cylindrospermopsin. Environ. Toxicol..

[20] Turner P.C., Gammie A.J., Hollinrake K., Codd G.A. (1990). Pneumonia associated with contact with cyanobacteria. Br. Med. J..

[21] Jochimsen E.M., Carmichael W.W., An J.S., Cardo D.M. (1998). Liver failure and death after exposure to microcystins at a hemodialysis center in Brazil. N. Engl. J. Med..

[22] Pouria S., de Andrade A., Barbosa J., Cavalcanti R.L., Barreto V.T.S., Ward C.J., Preiser W., Poon G.K., Neild G.H., Codd G.A. (1998). Fatal microcystin intoxication in haemodialysis unit in Caruaru, Brazil. Lancet.

[23] Azevedo S.M.F.O., Carmichael W.W., Jochimsen E.M., Rinehard K.L., Lau S., Shaw G.R., Eaglesham G.K. (2002). Human Intoxication by microcystin during renal dialysis treatment in Caruaru—Brazil. Toxicology.

[24] Hu Y., Chen J., Fan H., Xie P., He J. (2016). A review of the neurotoxicity of microcystins. Environ. Sci. Pollut. Res..

[25] Aráoz R., Molgo J., Tandeau de Marsac N. (2010). Neurotoxic cyanobacterial toxins. Toxicon.

[26] Bruno M., Ploux O., Metcalf J.S., Mejean A., Pawlik-Skowronska B., Furey A., Meriluoto J., Spoof L., Codd G.A. (2017). Anatoxin-a, homoanatoxin-a, and natural analogues. Handbook of Cyanobacterial Monitoring and Cyanotoxin Analysis.

[27] Carmichael W.W., Biggs D.F., Peterson M.A. (1979). Pharmacology of anatoxin-a, produced by the freshwater cyanophyte *Anabaena flos-aquae* NRC-44-1. Toxicon.

[28] Soliakov L., Gallagher T., Wonnacott S. (1995). Anatoxin-a evoked [3H] dopamine release from rat striatial synaptosomes. Neuropharmacology.

[29] Adeyemo O.M., Siren A.L. (1992). Cardio-respiratory changes and mortality in the conscious rat induced by (+) and (+/-)-anatoxin-a. Toxicon.

[30] Schmitt C., Torrents R., Domange B., Simon N., de Haro L. (2019). Cerebellar syndrome associated with ingestion of Mediterranean Microcosmus: A French case series. Clin. Toxicol..

[31] Biré R., Bertin T., Dom I., Hort V., Schmitt C., Diogène J., Lemée R., de Haro L., Nicolas M. (2020). First evidence of the presence of anatoxin-a in sea figs associated with human poisonings in France. Mar. Drugs.

[32] Krienitz L., Ballot A., Kotut K., Wiegand C., Putz S., Metcalf J.S., Codd G.A., Pflugmacher S. (2003). Contribution of hot spring cyanobacteria to the mysterious deaths of Lesser Flamingos at Lake Bogoria, Kenya. FEMS Microbiol. Ecol..

[33] Wiese M., D’Agostino P.M., Mihali T.K., Moffitt M.C., Neilan B.A. (2010). Neurotoxic alkaloids: Saxitoxin and its analogs. Mar. Drugs.

[34] O’Neill K., Musgrave I.F., Humpage A. (2016). Low dose extended exposure to saxitoxin and its potential neurodevelopmental effects: A review. Environ. Toxicol. Pharmacol..

[35] Ballot A., Bernard C., Fastner J., Meriluoto J., Spoof L., Codd G.A. (2017). Saxitoxin and analogues. Handbook of Cyanobacterial Monitoring and Cyanotoxin Analysis.

[36] Cusick K.D., Sayler G.S. (2013). An overview on the marine neurotoxin, saxitoxin: Genetics, molecular targets, methods of detection and ecological functions. Mar. Drugs.

[37] Lipkind G.M., Fozzard H.A. (1994). A structural model of the tetrodotoxin and saxitoxin binding site of the Na^+^ channel. Biophys. J..

[38] Llewellyn L.E. (2006). Saxitoxin, a toxic marine natural product that targets a multitude of receptors. Nat. Prod. Rep..

[39] Fiore M.F., de Lima S.T., Carmichael W.W., McKinnie S.M.K., Chekan J.R., Moore B.S. (2020). Guanitoxin, re-naming a cyanobacterial organophosphate toxin. Harmful Algae.

[40] Mahmood N.A., Carmichael W.W. (1986). The pharmacology of anatoxin-a(s), a neurotoxin produced by the freshwater cyanobacterium *Anabaena flos-aquae*. Toxicon.

[41] Devic E., Li D., Dauta A., Henriksen P., Codd G.A., Marty J.-L., Fournier D. (2002). Detection of anatoxin-a(S) in environmental samples by using a biosensor with engineered acetylcholinesterases. Appl. Environ. Microbiol..

[42] Metcalf J.S., Bruno M., Meriluoto J., Spoof L., Codd G.A. (2017). Anatoxin-a(S). Handbook of Cyanobacterial Monitoring and Cyanotoxin Analysis.

[43] Metcalf J.S., Richer R., Cox P.A., Codd G.A. (2012). Cyanotoxins in desert environments may present a risk to human health. Sci. Total Environ..

[44] Nishiwaki-Matsushima R., Ohta T., Nishiwaki S., Suganuma M., Kohyama K., Ishikawa T., Carmichael W.W., Fujiki H. (1992). Liver tumor promotion by the cyanobacterial cyclic peptide toxin microcystin-LR. J. Cancer Res. Clin. Oncol..

[45] Ohta T., Sueoka E., Lida N., Komori A., Suganuma M., Nishiwaki R., Tatematsu M., Kim S.J., Carmichael W.W., Fujiki H. (1994). Nodularin, a potent inhibitor of protein phosphatases 1 and 2A, is a new environmental carcinogen in male F344 rat liver. Cancer Res..

[46] Ueno Y., Nagata S., Tsutsumi T., Hasegawa A., Watanabe M.F., Park H.D., Chen G.C., Chen G., Yu S.Z. (1996). Detection of microcystins, a blue-green algal hepatotoxin, in drinking water sampled in Haimen and Fusui, endemic areas of primary liver cancer in China, by highly sensitive immunoassay. Carcinogenesis.

[47] Falconer I.R., Humpage A.R. (2001). Preliminary evidence for in vivo tumour initiation by oral administration of extracts of the blue-green alga Cylindrospermopsis raciborskii containing the toxin cylindrospermopsin. Environ. Toxicol..

[48] Herrup K., Neve R., Ackerman S.L., Copani A. (2004). Divide and die: Cell cycle events as triggers of nerve cell death. J. Neurosci..

[49] Caller T.A., Doolin J.W., Haney J.F., Murby A.J., West K.G., Farrar H.E., Ball A., Harris B.T., Stommel E.W. (2009). A cluster of amyotrophic lateral sclerosis in New Hampshire: A possible role for toxic cyanobacteria blooms. Amyotroph. Lateral Scler..

[50] Cox P.A., Kostrzewa R.M., Guillemin G.J. (2018). BMAA and neurodegenerative illness. Neurotox. Res..

[51] Reed D., Labarthe D., Chen K.M., Stallones R.A. (1987). Cohort study of amyotrophic lateral sclerosis and Parkinsonism/dementia on Guam and Rota. Am. J. Epidemiol..

[52] Vega A., Bell E.A. (1967). α-amino-β-methylaminopropionic acid, a new amino acid from seeds of *Cycas circinalis*. Phytochemistry.

[53] Polsky F.I., Nunn P.B., Bell E.A. (1972). Distribution and toxicity of alpha-amino-beta-methylaminopropionic acid. Fed. Proc..

[54] Cox P.A., Banack S.A., Murch S.J., Rasmussen U., Tien G., Bidigare R.R., Metcalf J.S., Morrison L.F., Codd G.A., Bergman B. (2005). Diverse taxa of cyanobacteria produce β-N-methylamino-L-alanine, a neurotoxic amino acid. Proc. Natl. Acad. Sci. USA.

[55] Downing S., Banack S.A., Metcalf J.S., Cox P.A., Downing T.G. (2011). Nitrogen starvation of cyanobacteria results in the production of β-N-methylamino-L-alanine. Toxicon.

[56] Jiang L., Eriksson J., Lage S., Jonasson S., Shams S., Mehine M., Ilag L.L., Rasmussen U. (2014). Diatoms: A novel source for the neurotoxin BMAA in aquatic environments. PLoS ONE.

[57] Violi J.P., Facey J.A., Mitrovic S.M., Colville A., Rodgers K.J. (2019). Production of β-methylamino-L-alanine (BMAA) and its isomers by freshwater diatoms. Toxins.

[58] Nunn P.B., Codd G.A. (2019). Environmental distribution of the neurotoxin L-BMAA in *Paenibacillus* species. Toxicol. Res..

[59] Lage S., Costa P.R., Moita T., Eriksson J., Rasmussen U., Rydberg S.J. (2014). BMAA in shellfish from two Portuguese water bodies suggests the marine dinoflagellate Gymnodinium catenatum as a potential BMAA source. Aquat. Toxicol..

[60] Metcalf J.S., Banack S.A., Wesel R.A., Lester M., Pim J.G., Cassani J.R., Cox P.A. (2021). Toxin analysis of freshwater cyanobacterial and marine harmful algal blooms on the west coast of Florida and implications for estuarine environments. Neurotox. Res..

[61] Murch S.J., Cox P.A., Banack S.A., Steele J.C., Sacks O.W. (2004). Occurrence of β-methylamino-L-alanine (BMAA) in ALS/PDC patients from Guam. Acta Neurol. Scand..

[62] Pablo J., Banack S.A., Cox P.A., Johnson T.E., Papapetropoulos S., Bradley W.G., Buck A., Mash D.C. (2009). Cyanobacteial neurotoxin BMAA in ALS and Alzheimer’s disease. Acta Neurol. Scand..

[63] Cox P.A., Davis D.A., Mash D.C., Metcalf J.S., Banack S.A. (2016). Dietary exposure to an environmental toxin triggers neurofibrillary tangles and amyloid deposits in the brain. Proc. R. Soc. B.

[64] Davis D.A., Cox P.A., Banack S.A., Lecusay P.D., Garamszegi S.P., Hagan M.J., Powell J.T., Metcalf J.S., Palmour R.M., Beierschmitt A. (2020). L-serine reduces spinal cord pathology in a vervet model of preclinical ALS/MND. J. Neuropathol. Exp. Neurol..

[65] Levine T.D., Miller R.G., Bradley W.G., Moore D.H., Saperstein D.S., Flynn L.E., Katz J.S., Forshew D.A., Metcalf J.S., Banack S.A. (2017). Phase I clinical trial of safety of L-serine for ALS patients. Amytotroph. Lat. Scler. Front. Degen..

[66] Torbick N., Ziniti B., Stommel E., Linder E., Andrew A., Caller T., Haney J., Bradley W., Henegan P.L., Shi X. (2018). Assessing cyanobacterial harmful algal blooms as risk factors for amyotrophic lateral sclerosis. Neurotox. Res..

[67] Metcalf J.S., Lobner D., Banack S.A., Cox G.A., Nunn P.B., Wyatt P.B., Cox P.A. (2017). Analysis of BMAA enantiomers in cycads, cyanobacteria, and mammals: In vivo formation and toxicity of D-BMAA. Amino Acids.

[68] Schneider T., Simpson C., Desai P., Tucker M., Lobner D. (2020). Neurotoxicity of isomers of the environmental toxin L-BMAA. Toxicon.

[69] Salomonsson M.L., Fredriksson E., Alfjorden A., Hedeland M., Bondesson U. (2015). Seafood sold in Sweden contains BMAA: A study of free and total concentrations with UHPLC-MS/MS and dansyl chloride derivatization. Toxicol. Rep..

[70] Metcalf J.S., Codd G.A. (2020). Co-occurrence of cyanobacteria and cyanotoxins with other environmental health hazards: Impacts and implications. Toxins.

[71] Thomas N.J., Meteyer C.U., Sileo L. (1998). Epizootic vacuolar myelinopathy of the central nervous system of Bald Eagles (*Haliaeetus leucocephalus*) and American Coots (*Fulica americana*). Vet. Path..

[72] Wilde S.B., Murphy T.M., Hope C.P., Habrun S.K., Kempton J., Birrenkott A., Wiley F., Bowerman W.W., Lewitus A.J. (2005). Avian vacuolar myelinopathy linked to exotic aquatic plants and a novel cyanobacterial species. Env. Toxicol..

[73] Wilde S.B., Johansen J.R., Wilde H.D., Jiang P., Bartelme B., Haynie R.S. (2014). *Aetokthonos hydrillicola gen et sp. nov.*: Epiphytic cyanobacteria on invasive aquatic plants implicated in avian vacuolar myelinopathy. Phytotaxa.

[74] Brienlinger S., Phillips T.J., Haram B.N., Mares J., Yerena J.A.M., Hrouzek P., Sobotka R., Henderson W.M., Schmieder P., Williams S.M. (2021). Hunting the eagle killer: A cyanobacterial neurotoxin causes vacuolar myelinopathy. Science.

[75] Spoof L., Catherine A., Meriluoto J., Spoof L., Codd G.A. (2017). Appendix 3, tables of microcystins and nodularins. Handbook of Cyanobacterial Monitoring and Cyanotoxin Analysis.

[76] Catherine A., Bernard C., Spoof L., Bruno M., Meriluoto J., Spoof L., Codd G.A. (2017). Microcystins and nodularins. Handbook of Cyanobacterial Monitoring and Cyanotoxin Analysis.

[77] MacKintosh C., Beattie K.A., Klumpp S., Cohen P., Codd G.A. (1990). Cyanobacterial microcystin-LR is a potent and specific inhibitor of protein phosphatases 1 and 2A from both mammals and higher plants. FEBS Letts..

[78] Hastie C.J., Borthwick E.B., Morrison L.F., Codd G.A., Cohen P.T.W. (2005). Inhibition of several protein phosphatases by a non-covalently interacting microcystin and a novel cyanobacterial peptide, nostocyclin. Biochim. Biophys. Acta.

[79] Arif M., Kazim S.F., Grunde-Iqbal I., Garruto R.M., Iqbal K. (2014). Tau pathology involves protein phosphatase 2A in Parkinsonism-dementia of Guam. Proc. Natl. Acad. Sci. USA.

[80] WHO (2020). Cyanobacterial Toxins: Microcystins. Background Document for Development of WHO Guidelines for Drinking-Water Quality and Guidelines for Safe Recreational Water Environments.

[81] Elersek T., Blaha L., Mazur-Marzec H., Schmidt W., Carmeli S., Meriluoto J., Spoof L., Codd G.A. (2017). Other cyanobacterial bioactive substances. Handbook of Cyanobacterial Monitoring and Cyanotoxin Analysis.

[82] Berman F.W., Gerwick W.H., Murray T.F. (1999). Antillatoxin and kalkitoxin, ichthyotoxins from the tropical cyanobacterium Lyngbya majuscula, induce distinct temporal patterns of NMDA receptor-mediated toxicity. Toxicon.

[83] Li W.I., Berman F.W., Okino T., Yokokawa F., Shiori T., Gerwick W.H., Murray T.F. (2001). Antillatoxin is a marine cyanobacterial toxin that potentially activates voltage-gated sodium channels. Proc. Natl. Acad. Sci. USA.

[84] Du X., Liu H., Yuan L., Wang Y., Ma Y., Wang R., Chen X., Losiewicz M.D., Guo H., Zhang H. (2019). The diversity of cyanobacterial toxins on structural characterization, distribution and identification: A systematic review. Toxins.

[85] Zhang F., Xu X., Li T., Liu Z. (2013). Shellfish toxins targeting voltage-gated sodium channels. Mar. Drugs.

[86] Florczyk M., Lakomiak A., Wozny M., Brzuzan P. (2010). Neurotoxicity of cyanobacterial toxins. Environ. Biotechnol..

[87] Byth S. (2014). Palm Island mystery disease. Med. J. Aust..

[88] Hawkins P., Runnegar M.C., Jackson A.B., Falconer I.R. (1985). Severe Hepatotoxicity Caused by the Tropical Cyanobacterium (Blue-Green Alga) *Cylindrospermopsis raciborskii* (Woloszynska) Seenaya and Subba Raju Isolated from a Domestic Water Supply Reservoir. Appl. Environ. Microbiol..

[89] Hinojosa M.G., Gutierrez-Praena D., Prieto A.I., Guzman-Guillen R., Jos A., Camean A.M. (2019). Neurotoxicity induced by microcystins and cylindrospermopsin: A review. Sci. Total Environ..

[90] Kiss T., Vehovsky A., Hiripi L., Kovács A., Vörös L. (2002). Membrane effects of toxins isolated from a cyanobacterium, *Cylindrospermopsis raciborskii* on identified molluscan neurones. Comp. Biochem. Physiol. Part C Toxicol. Pharmacol..

[91] Vehovsky A., Kovács A.W., Farkas A., Györi J., Szabó H., Vasas G. (2013). Pharmacological studies confirm neurotoxic metabolite(s) produced by the bloom-forming *Cylindrospermopsis racirborskii* in Hungary. Environ. Toxicol..

[92] Tasker L., Benachour N., Husk B., Cabana H., Gris D. (2016). Cyanotoxins at low doses induce apoptosis and inflammatory effects in murine brain cells: Potential Implications for neurodegenerative diseases. Toxicol. Rep..

[93] Sano T., Usui T., Udeka K., Osada H., Kaya K. (2001). Isolation of new protein phosphatase inhibitors from two cyanobacteria species, *Planktothrix* spp.. J. Nat. Prod..

[94] Sano T., Takagi H., Morrison L.F., Metcalf J.S., Codd G.A., Kaya K. (2005). Leucine aminopeptidase M inhibitors, cyanostatin A and B, isolated from cyanobacterial water blooms in Scotland. Phytochemistry.

[95] Gemma S., Molteni M., Rossetti C. (2016). Lipopolysaccharides in cyanobacteria: A brief overview. Adv. Microbiol..

[96] Lee S., Kim J.-H., Seo J.-W., Han H.-S., Lee W.-H., Mori K., Nakao K., Barasch J., Suk K. (2011). Lipocalin-2 is a chemokine inducer in the central nervous system. J. Biol. Chem..

[97] Hawkins B.T., Davis T.P. (2005). The blood-brain barrier/neurovascular unit in health and disease. Pharmacol. Rev..

[98] Bolla K.I., Rothman R., Cadet J.L. (1999). Dose-related neurobehavioral effects of chronic cocaine use. J. Neuropsychiatry Clin. Neurosci..

[99] Clarke P.B.S., Rueben M. (1996). Release of [^3^H]-noradrenaline from rat hippocampal synaptosomes by nicotine: Mediation by different nicotinic receptor subtypes from striatal [^3^H]-dopamine release. Br. J. Pharmacol..

[100] Campos F., Durán R., Vidal L., Faro L.R.F., Alfonso M. (2006). In vivo effects of the anatoxin-a on striatal dopamine release. Neurochem. Res..

[101] Aronstam R.S., Witkop B. (1981). Anatoxin-a interactions with cholinergic synaptic molecules. Proc. Natl. Acad. Sci. USA.

[102] Tega Y., Yamazaki Y., Akanuma S.-I., Kubo Y., Hosoya K.-I. (2018). Impact of nicotine transport across the blood-brain barrier: Carrier mediated transport of nicotine and interaction with central nervous system drugs. Biol. Pharm. Bull..

[103] Pappano A.J., Katzung B.G., Masters S.B., Trevor A.J. (2012). Cholinergic-Activating & Cholinesterase-Inhibiting Drugs. Basic & Clinical Pharmacology.

[104] Costa L.G., Klaassen C.D. (2008). Toxic effects of pesticides. Casarett and Doull’s Toxicology, The Basic Science of Poisons.

[105] Webster L.R., McKenzie G.H., Moriarty H.T. (2002). Organophosphate-based pesticides and genetic damage implicated in bladder cancer. Cancer Gen. Cytogen..

[106] Wang A., Costello S., Cockburn M., Zhang X., Bronstein J., Ritz B. (2011). Parkinson’s disease risk from ambient exposure to pesticides. Eur. J. Epidemiol..

[107] Wang A., Cockburn M., Ly T.T., Bronstein J.M., Ritz B. (2014). The association between ambient exposure to organophosphates and Parkinson’s disease risk. Occup. Environ. Med..

[108] Schneider Medeiros M., Reddy S.P., Socal M.P., Schumacher-Schuc A.F., Rieder C.R.M. (2020). Occupational pesticide exposure and the risk of death in patients with Parkinson’s disease: An observational study in southern Brazil. Environ. Health.

[109] Kamel F., Umbach D.M., Bedlack R.S., Richards M., Watson M., Alavanja M.C., Blair A., Hoppin J.A., Schmidt S., Sandler D.P. (2012). Pesticide exposure and amyotrophic lateral sclerosis. Neurotoxicology.

[110] Merwin S.J., Obis T., Nunez Y., Re D.B. (2017). Organophosphate neurotoxicity to the voluntary motor system on the trail of environment-caused amyotrophic lateral sclerosis: The known, the misknown and the unknown. Arch. Toxicol..

[111] Lotti M., Moretto A. (2005). Organophosphate-induced delayed polyneuropathy. Toxicol. Rev..

[112] Balbuena P., Li W., Magnin-Bissel G., Meldrum J.B., Ehrich M. (2010). Comparison of two blood-brain barrier in vitro systems: Cytotoxicity and transfer assessments of malathion/oxon and lead acetate. Toxicol. Sci..

[113] Balbuena P., Li W., Ehrich M. (2011). Assessments of tight junctional proteins occludin, claudin 5 and scaffold proteins ZO_1_ and ZO_2_ in endothelial cells of the rat blood-brain barrier: Cellular responses to neurotoxicants malathion and lead acetate. Neurotoxicology.

[114] Ravid O., Goldman S.E., Macheto D., Bresler Y., De Oliveira R.I., Liraz-Zaltsman S., Gosselet F., Dehouck L., Beeri M.S., Cooper I. (2018). Blood-brain barrier cellular responses toward organophosphates: Natural compensatory processes and exogenous interventions to rescue barrier properties. Front. Cell. Neurosci..

[115] Andrinolo D., Michea L.F., Lagos N. (1999). Toxic effects, pharmacokinetics and clearance of saxitoxin, a component of paralytic shellfish poison (PSP), in cats. Toxicon.

[116] Cianca R.C.C., Pallares M.A., Barbosa R.D., Adan L.V., Martins J.M.L., Gago-Martínez A. (2007). Application of precolumn oxidation HPLC method with fluorescence detection to evaluate saxitoxin levels in discrete brain regions of rats. Toxicon.

[117] Cervantes C.R.C., Durán R., Faro L.F., Alfonso P.M. (2009). Effects of systemic administration of saxitoxin on serotonin levels in some discrete rat brain regions. Med. Chem..

[118] Melnikova D.I., Khotimchenko Y.S., Magarlamov T.Y. (2018). Addressing the issue of tetrodotoxin targeting. Mar. Drugs.

[119] Fischer W.J., Altheimer S., Cattori V., Meier P.J., Dietrich D.R., Hagenbuch B. (2005). Organic anion transporting polypeptides expressed in liver and brain mediated uptake of microcystin. Toxicol. Appl. Pharmacol..

[120] Furstein D., Holst K., Fischer A., Dietrich D.R. (2009). Oatp-associated uptake and toxicity of microcystins in primary murine whole brain cells. Toxicol. Appl. Pharmacol..

[121] Furstein D., Kleinteich J., Heussner A.H., Stemmer K., Dietrich D.R. (2010). Investigation of microcystin congener-dependent uptake into primary murine neurons. Environ. Health Perspect..

[122] Ward C.J., Codd G.A. (1999). Comparative toxicity of four microcystins of different hydrophobicities to the protozoan, *Terahymena Pyriformis*. J. Appl. Microbiol..

[123] Guzmán-Guillén R., Manzano I.L., Moreno I.M., Ortega A.I.P., Moyano R., Blanco A., Cameán A.M. (2015). Cylindrospermopsin induces neurotoxicity in tilapia fish (Oreochromis niloticus) exposed to Aphanizomenon ovalisporum. Aquat. Toxicol..

[124] Banks W.A. (2009). Characteristics of compounds that cross the blood-brain barrier. BMC Neurol..

[125] Metcalf J.S., Young F.M., Codd G.A. (2017). Performance assessment of a cylindrospermopsin ELISA with purified compounds and cyanobacterial extracts. Environ. Forensics.

[126] Xie X., Basile M., Mash D.C. (2013). Cerebral uptake and protein incorporation of cyanobacterial toxin beta-N-methylamino-L-alanine. Neuroreport.

[127] Davis D.A., Mondo K., Stern E., Annor A.K., Murch S.J., Coyne T.M., Brand L.E., Niemeyer M.E., Sharp S., Bradley W.G. (2019). Cyanobacterial neurotoxin BMAA and brain pathology in stranded dolphins. PLoS ONE.

[128] Smith Q.R., Nagura H., Takada Y., Duncan M.W. (1992). Facilitated transport of the neurotoxin, β-*N*-methylamino-L-alanine, across the blood brain barrier. J. Neurochem..

[129] Nunn P.B. (2009). Three phases of research on β-N-methylamino-L-alanine (BMAA)—A neurotoxic amino acid. Amyotroph. Lateral Scler..

[130] Bell E.A., Watson A.A., Nash R.J. (2008). Non-protein amino acids: A review of the biosynthesis and taxonomic significance. Nat. Prod. Comm..

[131] Dunlop R.A., Main B.J., Rodgers K.J. (2015). The deleterious effects of non-protein amino acids from desert plants on human and animal health. J. Arid Environ..

[132] Narahashi T., Haas H.G., Therrien E.F. (1967). Saxitoxin and tetrodotoxin: Comparison of nerve blocking mechanism. Science.

[133] Zhang X., Nordberg A. (1993). The competition of (-)-[3H]nicotine binding by the enantiomers of nicotine, nornicotine and anatoxin-a in membranes and solubilized preparations of different brain regions of rat. Naunyn-Schmiedeberg. Arch. Pharmacol..

[134] Wiess J.H., Christine C.W., Choi D.W. (1989). Bicarbonate dependence of glutamate receptor activation by β-*N*-methylamino-L-alanine: Channel recording and study with related compounds. Neuron.

[135] Allen C.N., Omelchenko L., Ross M., Spencer P. (1995). The neurotoxin, β-*N*-methylamino-L-alanine (BMAA) interacts with the strychnine-insensitive glycine modulatory site of the *N*-methyl-D-aspartate receptor. Neuropharmacology.

[136] Metcalf J.S., Hiskia A., Kaloudis T., Meriluoto J., Spoof L., Codd G.A. (2017). Protein phosphatase inhibition assays. Handbook of Cyanobacterial Monitoring and Cyanotoxin Analysis.

[137] Dunlop R.A., Cox P.A., Banack S.A., Rodgers K.J. (2013). The non-protein amino acid BMAA is misincorporated in place of L-serine causing protein misfolding and aggregation. PLoS ONE.

[138] Lee J.W., Beebe K., Nangle L.A., Jang J., Longo-Guess C.M., Cook S.A., Davisson M.T., Sundberg K.P., Schimmel P., Ackerman S.L. (2006). Editing-defective tRNA synthetase causes protein misfolding and neurodegeneration. Nature.

[139] Bláha L., Cameán V., Gutiérrez-Praena D., Jos A., Marie B., Metcalf J.S., Pichardo S., Puerto M., Törökné A., Vasas G., Merilluoto J., Spoof L., Codd G.A. (2017). Bioassay use in the field of toxic cyanobacteria. Handbook of Cyanobacterial Monitoring and Cyanotoxin Analysis.

[140] Negri A., Llewellyn L. (1998). Comparative analyses by HPLC and the sodium channel and saxiphilin ^3^H-saxitoxin receptor assays for paralytic shellfish toxins in crustaceans and molluscs from tropical north west Australia. Toxicon.

[141] Weller M.G. (2013). Immunoassays and biosensors for the detection of cyanobacterial toxins in water. Sensors.

[142] Haddad S.P., Bobbitt J.M., Taylor R.B., Lovin L.M., Conkle J.L., Chambliss C.K., Brooks B.W. (2019). Determination of microcystins, nodularin, anatoxin-a, cylindrospermopsin, and saxitoxin in water and fish tissue using isotope dilution liquid chromatography tandem mass spectrometry. J. Chromatogr. A.

[143] Dörr F.A., Rodríguez V., Molica R., Henriksen P., Krock B., Pinto E. (2010). Methods for detection of anatoxin-a(s) by liquid chromatography coupled to electrospray ionization-tandem mass spectrometry. Toxicon.

[144] Fernandes K.A., Ferraz H.G., Vereau F., Pinto E. (2020). Availability of guanitoxin in water samples containing *Sphaerospermopsis torques-reginae* cells submitted to dissolution tests. Pharmaceuticals.

[145] Suzuki H., Machii K. (2014). Comparison of toxicity between saxitoxin and decarbamoyl saxitoxin in the mouse bioassay for paralytic shellfish poisoning toxins. J. Vet. Med. Sci..

[146] Lahti K., Ahtiainen J., Rapala J., Sivonen K., Niemelä S.I. (1995). Assessment of rapid bioassays for detecting cyanobacterial toxicity. Letts. Appl. Microbiol..

[147] Ferrão-Filho A.D.S., Soares M.C.S., de Magalhães V.F., Azevedo S.M. (2010). A rapid bioassay for detecting saxitoxins using a Daphnia acute toxicity test. Environ. Pollut..

[148] Svircev Z., Drobac D., Tokodi N., Vidovic M., Simeunovic J., Miladinov-Mikov M., Baltic V. (2013). Epidemiology of primary liver cancer in Serbia and possible connection with cyanobacterial blooms. J. Environ. Sci. Health C Environ. Carcinog. Ecotoxicol. Rev..

[149] Turner A.D., Dhanji-Rapkova M., Dean K., Milligan S., Hamilton M., Thomas J., Poole C., Haycock J., Spelman-Marriott J., Watson A. (2018). Fatal canine intoxications linked to the presence of saxitoxins in stranded marine organisms following winter storm activity. Toxins.

[150] Suarez-Isla B.A., Gopalakrishnakone P., Haddad V., Kem W.R., Tubaro A., Kim E. (2015). Saxitoxin and other paralytic toxins: Toxicological profile. Marine and Freshwater Toxins.

[151] Elleman A.V., Devienne G., Makinson C.D., Haynes A.L., Huguenard J.R., Du Bois J. (2021). Precise spatiotemporal control of voltage-gated sodium channels by photocaged saxitoxin. Nat. Comm..

[152] Watanabe R., Kanamori M., Yoshida H., Okumura Y., Uchida H., Matsushima R., Oikawa H., Suzuki T. (2019). Development of ultra-performance liquid chromatography with post-column fluorescent derivatization for the rapid detection of saxitoxin analogues and analysis of bivalve monitoring samples. Toxins.

[153] Gibble C.M., Kudela R.M., Knowles S., Bodenstein B., Lefebvre K.A. (2021). Domoic acid and saxitoxin in the United States between 2007 and 2018. Harmful Algae.

[154] Liu L., Chen J., He X., Hao S., Lian Z., Wang B. (2020). First determination of extracellular paralytic shellfish poisoning toxins in the culture medium of toxigenic dinoflagellates by HILIC-HRMS. Ecotoxicol. Environ. Saf..

[155] Arman T., Clarke J.D. (2021). Microcystin toxicokinetics, molecular toxicology and pathophysiology in preclinical rodent models and humans. Toxins.

[156] Dreher T.W., Collart L.P., Mueller R.S., Halsey K.H., Bildfell R.J., Schreder P., Sobhakumari A., Ferry R. (2019). *Anabaena/Dolichospermum* as the source of lethal microcystin levels responsible for a large cattle toxicosis event. Toxicon X.

[157] McCain S., Sim R.R., Howerth E.W., Aschenbroich S., Kirejczyk S.G.M., McHale B., Jerry C., Kottwitz J.J., Wilson A.E., McManamon R. (2020). Myonecrosis and death due to presumed microcystin toxicosis in American White Pelicans (*Pelecanus erythrorhyncos*). J. Zoo Wildlife Med..

[158] Swe T., Miles C.O., Cerasino L., Mjelde M., Kleiven S., Ballot A. (2021). *Microcystis, Rhaphidiopsis raciborskii* and *Dolichospermum smithii*, toxin producing and non-toxigenic cyanobacteria in Yezin Dam, Myanmar. Limnologica.

[159] Foss A.J., Aubel M.T., Gallagher B., Mettee N., Miller A., Fogelson S.B. (2019). Diagnosing microcystin intoxication of canines: Clinicopathological indications, pathological characteristics, and analytical detection in postmortem and antemortem samples. Toxins.

[160] Massey I.Y., Wu P., Wei J., Luo J., Ding P., Wei H., Yang F. (2020). A mini-review on detection methods of microcystins. Toxins.

[161] Christensen V.G., Khan E. (2020). Freshwater neurotoxins and concerns for human, animal and ecosystem health: A review of anatoxin-a and saxitoxin. Sci. Total Environ..

[162] Bauer F., Fastern J., Bartha-Dima B., Breuer W., Falkenau A., Mayer C., Raeder U. (2020). Mass occurrence of anatoxin-a and dihydroanatoxin-a-producing *Tychonema* sp. in mesotrophic reservoir Mandichosee (River Lech, Germany) as a cause of neurotoxicosis in dogs. Toxins.

[163] Ballot A., Krienitz L., Kotut K., Wiegand C., Metcalf J.S., Codd G.A., Pflugmacher S. (2004). Cyanobacteria and cyanobacterial toxins in three alkaline Rift Valley lakes of Kenya- Lakes Bogoria, Nakuru and Elmenteita. J. Plank. Res..

[164] LeDuc J.F., Christensen V.G., Maki R.P. (2020). Rapid-assessment test strips: Effectiveness for cyanotoxin monitoring in a northern temperate lake. Lake Reserv. Manag..

[165] Jaramillo M., O’Shea K.E. (2019). Analytical methods for assessment of cyanotoxin contamination in drinking water sources. Curr. Opin. Environ. Sci. Health.

[166] Bishop S.L., Murch S.J. (2020). A systematic review of analytical methods for the detection and quantification of β-*N*-methylamino-L-alanine (BMAA). Analyst.

[167] Froscio S.M., Humpage A.R., Burcham P.C., Falconer I.R. (2003). Cylindrospermopsin-induced protein synthesis inhibition and its dissociation from acute toxicity in mouse hepatocytes. Environ. Toxicol..

[168] Hiskia A., Spoof L., Kaloudis T., Meriluoto J., Meriluoto J., Spoof L., Codd G.A. (2017). Determination of cyanotoxins by high performance liquid chromatography with photodiode array. Handbook of Cyanobacterial Monitoring and Cyanotoxin Analysis.

[169] Romera-García E., Helmus R., Ballesteros-Gómez A., Visser P.M. (2021). Multi-class determination of intracellular and extracellular cyanotoxins in freshwater samples by ultra-high performance liquid chromatography coupled to high resolution mass spectrometry. Chemosphere.

[170] Reveillon D., Sechet V., Hess P., Amzil Z. (2016). Production of BMAA and DAB by diatoms (*Phaeodactylum tricornutum*, *Chaetoceros* sp., *Chaetoceros calcitrans* and, *Thalassiosira pseudonana*) and bacteria isolated from a diatom culture. Harmful Algae.

[171] Banack S.A., Murch S.J. (2009). Multiple neurotoxic items in the Chamorro diet link BMAA with ALS/PDC. Amyotroph. Lateral Scler..

[172] Bell E.A. (2009). The discovery of BMAA, and examples of biomagnification and protein incorporation involving other non-protein amino acids. Amytroph. Lateral Scler..

[173] Jungblut A.D., Wilbraham J., Banack S.A., Metcalf J.S., Codd G.A. (2018). Microcystins, BMAA and BMAA isomers in 100-year-old Antarctic cyanobacterial mats collected during Captain R.F. Scott’s Discovery Expedition. Eur. J. Phycol..

[174] Karamyan V.T., Speth R.C. (2008). Animal models of BMAA neurotoxicity: A critical review. Life Sci..

[175] Dunlop R.A., Banack S.A., Bishop S.L., Metcalf J.S., Murch S.J., Davis D.A., Stommel E.W., Karlsson O., Brittebo E.B., Chatziefthimiou A.D. (2021). Is exposure to BMAA a risk factor for neurodegenerative diseases? A response to a critical review of the BMAA hypothesis. Neurotox. Res..

[176] Pietsch C., Wiegand C., Amé M.V., Nicklisch A., Wunderlin D., Pflugmacher S. (2001). The effects of a cyanobacterial crude extract on different aquatic organisms: Evidence for cyanobacterial toxin modulating factors. Environ. Toxicol..

[177] Falconer I.R., Burch M.D., Steffensen D.A., Choice M., Coverdale O.R. (1994). Toxicity of the blue-green alga (cyanobacterium) *Microcystis aeruginosa* in drinking water to growing pigs, as an animal model for human injury and risk assessment. Environ. Toxicol. Water Qual..

[178] Fitzgeorge R.B., Clark S.A., Keevil C.W., Codd G.A., Jefferies T.M., Keevil C.W., Potter C. (1994). Routes of intoxication. Detection Methods for Cyanobacterial Toxins.

[179] Backer L.C., Carmichael W.W., Kirkpatrick B., Williams C., Irvin M., Zhou Y., Johnson T.B., Nierenberg K., Hill V.R., Kieszak S.M. (2008). Recreational exposure to low concentrations of microcystins during an algal bloom in a small lake. Mar. Drugs.

[180] Backer L.C., McNeel S.V., Barber T., Kirkpatrick B., Williams C., Irvin M., Zhou Y., Johnson T.B., Nierenberg K., Aubel M. (2010). Recreational exposure to microcystins during algal blooms in two California lakes. Toxicon.

[181] Banack S.A., Caller T., Henegan P., Haney J., Murby A., Metcalf J.S., Powell J.T., Cox P.A., Stommel E.A. (2015). Detection of cyanotoxins, β-*N*-methylamino-L-alanine and microcystins from a lake surrounded by cases of amyotrophic lateral sclerosis. Toxins.

[182] Sutherland J.W., Turcotte R.J., Molden E., Moriarty V., Kelly M., Aubel M., Foss A. (2021). The detection of airborne anatoxin-a (ATX) on glass fiber filters during a harmful algal bloom. Lake Reserv. Manage..

[183] Cox P.A., Richer R., Metcalf J.S., Banack S.A., Codd G.A., Bradley W.G. (2009). Cyanobacteria and BMAA exposure from desert dust: A possible link to sporadic ALS among Gulf War veterans. Amyotroph. Lateral Scler..

[184] Lucchini R.G., Doman D.C., Elder A., Veronesi B. (2012). Neurological impacts from inhalation of pollutants and the nose-brain connection. Neurotoxicology.

[185] Lindsay J., Metcalf J.S., Codd G.A. (2006). Protection against the toxicity of microcystin-LR and cylindrospermopsin in Artemia salina and Daphnia spp. by pre-treatment with cyanobacterial lipopolysaccharide (LPS). Toxicon.

[186] Martin R.M., Stallrich J., Bereman M.S. (2019). Mixture designs to investigate adverse effects upon co-exposure to environmental toxins. Toxicology.

